# Ensemble Methods for APS In-Flight Particle Temperature and Velocity Prediction Considering Torch Electrodes Ageing

**DOI:** 10.1007/s11666-022-01472-3

**Published:** 2023-01-06

**Authors:** K. R. Yu, C. V. Cojocaru, F. Ilinca, E. Irissou

**Affiliations:** National Research Council of Canada, AST, Boucherville, QC Canada

**Keywords:** atmospheric plasma spray (APS), particle temperature, particle velocity, process modeling, time-dependent modeling

## Abstract

The nonlinear relationship between the input process parameters and in-flight particle characteristics of the atmospheric plasma spray (APS) is of paramount importance for coating properties design and quality. It is also known that the ageing of torch electrodes affects this relationship. In recent years, machine learning algorithms have proven to be able to take into account such complex nonlinear interactions. This work illustrates the application of ensemble methods to predict the in-flight particle temperature and velocity during an APS process considering torch electrodes ageing. Experiments were performed to record simultaneously the input process parameters, the in-flight powder particle characteristics and the electrodes usage time. Random Forest (RF) and Gradient Boosting (GB) were used to rank and select the features for the APS process data recorded as the electrodes aged and the corresponding predictive models were compared. The time series aspect of the multivariate APS in-flight particle characteristics data is explored. Two strategies of time series embedding are considered. The first one simply embeds the attributes and the targets from the previous $n$ time segments considered without any modification; whereas the second strategy first performs differencing to make the time series stationary before embedding. For the present application, RF is found to be more suitable than GB since RF can predict both the in-flight particle velocity and temperature simultaneously, properly considering the interactions between the two targets. On the other hand, GB can only predict these two targets one at a time. The superior performance of both embedded predictive models and the feature rankings of them suggest that it is better to consider the APS data as time series for the in-flight particle characteristic prediction. In particular, it is demonstrated that it is advantageous to first make the time series stationary using the traditional differencing technique, even when modeling using RF.

## Introduction

The importance of the in-flight powder particle characteristics, such as the particle velocity and temperature, on the coating formation in the complex nonlinear atmospheric plasma spray (APS) process, is long well recognized. Since these characteristics cannot be measured during production, there is a great interest to predict these parameters to monitor and improve the spray process and coating quality.

The advancement of machine learning permits modeling such complex nonlinear relationships in a data-driven way. Guessasma et al. proposed to use artificial neural network (ANN) to develop an expert system for the prediction of the average spray particle velocity, temperature and diameter for better coating quality control (Ref 1). Input parameters, also referred to as attributes or predictors, including the arc current intensity, the argon and the hydrogen gas flow rate, were considered. The authors described their ANN development protocol in a follow-up article (Ref 2); in particular, they proposed a database enlargement procedure for the conditions when the number of experiments is insufficient to adequately train the ANN structure. Kanta et al. employed such protocol and developed an ANN to predict the in-flight particle temperatures and velocities to study the particle melting and dwell time with respect to the particle diameter (Ref 3). Sha expressed three concerns regarding such ANN development approach: first about the need and effectiveness of developing neural network models based on very few experimental data points, secondly about the database enlargement procedure and thirdly about the wide extrapolation based on the neural network models (Ref 4). Choudhury et al. borrowed the experimental data sets from (Ref 1) and suggested an alternative protocol to develop an ANN to predict the in-flight powder particle characteristics of an APS process (Ref 5); more specifically, the authors proposed to expand the experimental dataset using kernel regression instead and reported good model generalization. Later, Choudhury et al. employed extreme learning machine (ELM), a specific class of ANN, to construct a robust single hidden layer feed-forward neural network for the in-flight particle characteristics of the APS process (Ref 6). This approach reduced the training time and yielded stable performance with regard to the changes in the number of hidden layer neurons. Choudhury et al. further proposed to implement the ANN using a modular scheme (Ref 7). The scheme simplified the model structure and improved the generalization of the model overall. Liu et al. employed a nonlinear autoregressive exogenous (NLARX) model combined with the wavelet network to predict the in-flight particle characteristics of a mono-cathode plasma spray torch using a system identification approach (Ref 8). Compared with normal neural network, such approach could be more suitable for dynamical conditions. Recently, Zhu et al. proposed to employ convolutional neural network (CNN) to model the in-flight particle characteristics in atmospheric plasma spray (Ref 9). Proven to be successful for image processing, the CNN-based model can also describe the two-dimensional spray distributions along the plane of the substrate, in additional to the prediction of the average in-flight particle characteristic values as with an ANN. The reversed model can predict the required input control parameter given the two-dimensional sprayed particle distributions. More recently, Bobzin et al. proposed to use Residual Neural Network (ResNet) and Support Vector Machine (SVM) to provide fast estimations of the in-flight particle properties in APS. Numerical simulations are used to provide the training data. Both ResNet and SVM models are able to reduce the simulation time from 3 h down to a few seconds with accurate predictions for the average properties. Some other machine learning investigations for atmospheric plasma spray include process modeling, monitoring and control (Ref 10-12), coating characteristics prediction (Ref 13-16), and process optimization (Ref 17).

The majority of the predictive models proposed for APS in-flight particle characteristics are neural network based. There are however many other nonlinear predictive modeling techniques (Ref 18). In particular, decision tree-based ensemble methods, like random forest (RF) and gradient boosting (GB), have demonstrated their strong performance, often comparable to and sometimes even better than ANN (Ref 19, 20).

The essential idea behind ensemble methods is to combine the outputs from many simple models, referred to as base learners, to yield the final prediction. RF and GB are two popular ensemble methods; both use decision tree as their base learners. The two differs however in how the individual trees are constructed and added together. RF generates the trees by training them on subsets of data, both in terms of the observations and the attributes, randomly drawn from the full training set with replacement (Ref 21). The final prediction is an average of the results from all the generated trees. Since the generation of each tree does not depend on each other, the procedure is well suited to be executed in parallel. RF reduces the prediction variance. On the other hand, GB constructs each tree sequentially aiming to reduce the prediction error or the residue from the previous trees (Ref 22, 23). Subsampling in observations are generally considered, while subsampling in attributes may also be employed (Ref 24). GB reduces both the prediction variance and bias. Wolpert argues that without having substantive information about the modeling problem, there is no single model that will always do better than any other model (Ref 25). Could ensemble methods also predict well the in-flight particle characteristics?

It is well known that the ageing of torch electrodes greatly affect the relationship between the APS input parameters and the in-flight particle characteristics, in a time scale of hours. The same set of process inputs would expect to yield different in-flight particle characteristics using a brand new electrode pair as compared to a used one. Therefore, when modeling the in-flight particle characteristics with torch electrodes ageing considered, it may be imperative to consider the production data of APS as time series; where there is an ordered temporal component in the observations of the data. To the authors’ best knowledge, the usage time of torch electrodes (i.e. ageing) has not been previously considered along with other process parameters to predict the in-flight particle characteristics. Can better predictions be made if the production data of APS is considered as time series?

This work aims to explore the applicability of RF and GB, to predict and forecast the multivariate APS in-flight particle characteristics with the consideration of torch electrodes ageing as time series. In particular, two different time series modeling strategies are compared with the baseline approach. The paper is organized as follows: First, the electrode-wearing experiment is described, followed by the data preprocessing. Subsequently, the two time series modeling strategies are compared and discussed. The paper concludes that it is advantageous to consider the APS data as time series for the in-flight particle characteristic prediction. Also, it is beneficial to first make the time series stationary using the traditional differencing technique when modeling using RF for the present application.

## Experimental Procedures

APS experiments were performed to record simultaneously the spray process input parameters, the in-flight powder particle characteristics and the electrodes usage time. The experiment was carried out using a Metco 3 MB APS torch with a brand new pair of electrodes (model GE, nozzle diameter 5.54 mm), the electrodes were started 135 times and pursued until the torch could no longer sustain the plasma. The main process parameters (e.g. torch current intensity, voltage) were monitored and recorded at a sampling rate of 1 Hertz, using in-house built console equipment integrated into LabVIEW (National Instruments). Various spray time durations were considered for the torch usage so to emulate industrial coating spray production settings similar to those employed for thermal barrier coating (TBC) production. One single set of spray parameters, i.e. plasma-forming gases ${N}_{2}$ (50 lpm) as primary gas, ${H}_{2}$ (10 lpm) as auxiliary gas and a single TBC top coat powder (YSZ, Metco 204BN-S) deposited using a powder feed rate of 20 g/min with a constant carrier gas flow rate set at 6 lpm, and at 75 mm stand-off distance, was tested throughout the experiment. During the course of the experiment, it was aimed to maintain the net power of the torch constant by adjusting the torch current; where the net power is the raw power minus the heat transfer from the torch to the coolant (hereafter, referred to as torch cooling). An AccuraSpray (Tecnar, St-Bruno, Qc, Canada) diagnostic device was used to measure the in-flight particle temperature and velocity at defined time intervals (precision of the measurement given by the equipment i.e. plume relative intensity measurement 0.5% and plume geometry measurement 0.1 mm).

**Fig. 1:**
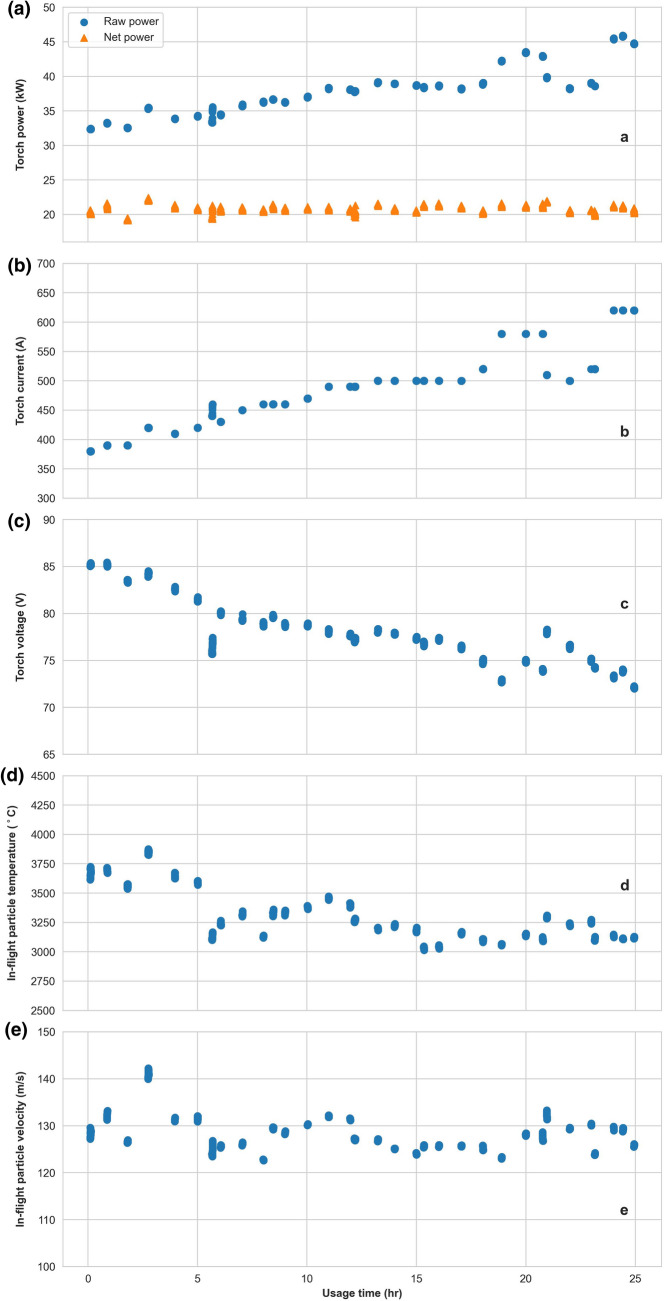
Torch power (a), current (b), voltage (c), as well as the corresponding in-flight particle temperature (d) and velocity (e) with respect to the usage time of the electrodes throughout the experiment. Throughout the experiment, it was aimed to maintain the net power of the torch constant by adjusting the torch current; where the net power is the raw power minus the torch cooling

**Fig. 2:**
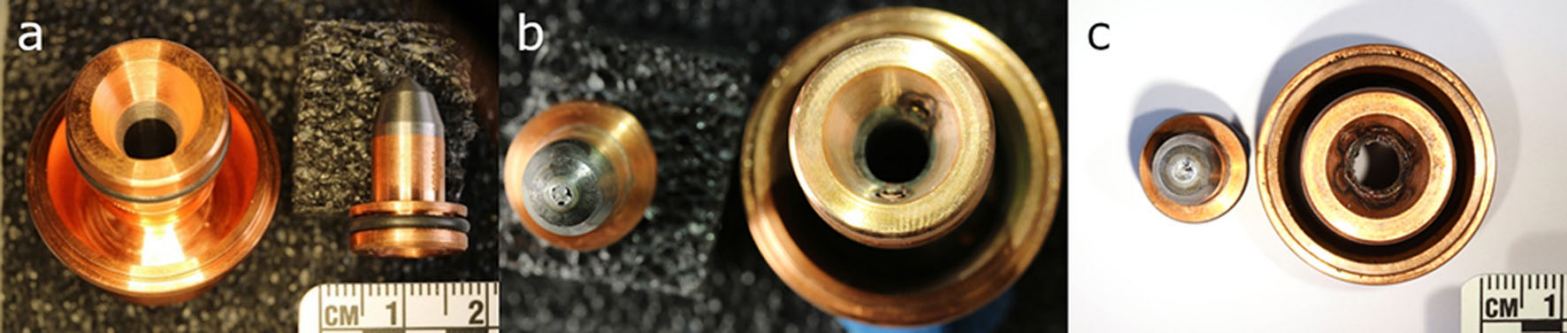
3 MB torch electrode pair used for the experiment. (a) shows the brand new electrode pair, (b) shows the electrode pair after 7 h of usage and finally (c) shows the electrode pair after 26 h of usage

**Fig. 3:**
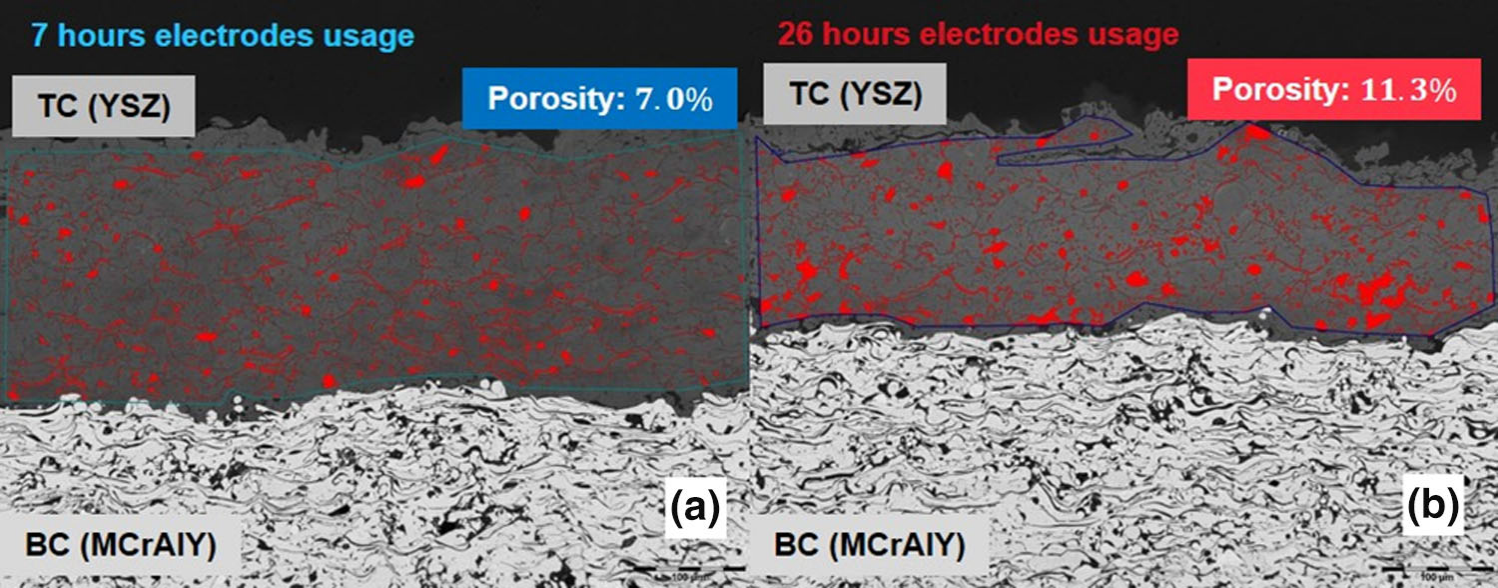
APS coating porosity. Increased porosity is observed in the coating when spraying with the electrodes used for 26 h (b) as compared to the ones used for 7 h (a). Here, TC refers to the YSZ top coat and BC refers to the MCrAlY bond coat

The electrodes began to show a weakness in sustaining a constant plasma plume and manifested plasma pulsations after about 26 h of usage. At that point, it was decided that the electrodes reached their end of life. The evolution of the torch current, voltage and power, and the corresponding in-flight particle velocity and temperature are shown in Fig. 1. Even though the spray condition is maintained constant during the experiment, with time the in-flight particle temperature shows an obvious decrease of about 500 °C from the beginning to the end of the experiment; whereas the in-flight particle velocity shows less reduction. The torch voltage is observed to decrease rather quickly within the first 5 h of usage, remains rather stable up to about 17 h of usage and shows a higher level of fluctuation at the end of usage time. The effect of electrode ageing on the in-flight particle characteristics can be clearly observed. Figure 2 shows the 3 MB torch electrode pair at the beginning, at 7 h of usage and at the end of the experiment. Figure 3 shows the porosity of the YSZ coating prepared at two different moments corresponding to different torch electrodes usage states; the coating prepared with the electrodes closer to the end of their lifetime (right) shows a higher level of porosity due to the electrode wearing.

## Predictive Modeling with Ensemble Methods

### Data Preprocessing

The data acquired from the different devices was first cleaned and then integrated based on the time stamp. For the data recorded from the APS controller (e.g. torch current intensity, voltage), only those observations when the plasma was on were considered. A total of about 2600 records with 8 attributes (including the torch voltage $V$ ($V$), the current $I$ ($A$), the input raw power ${P}_{raw}$ ($kW$), the net power ${P}_{net}$ ($kW$), the torch cooling ${q}_{cool}$ ($kW$), the flow rates of nitrogen ${Q}_{{N}_{2}}$ ($lpm$), hydrogen ${Q}_{{H}_{2}}$ ($lpm$) as well as, and the electrode usage time ${t}_{usage}$ (s)) and 2 targets (i.e. in-flight particle temperature ${T}_{p}$ (°C) and velocity ${v}_{p}$ ($m/s$)) were obtained right after the data fusion. All the attributes and the targets are normalized by subtracting their respective mean and then divided by their respective standard deviation. This common standardization can help to eliminate the possibility of numerical artifacts caused by different scaling during comparison.

**Fig. 4:**
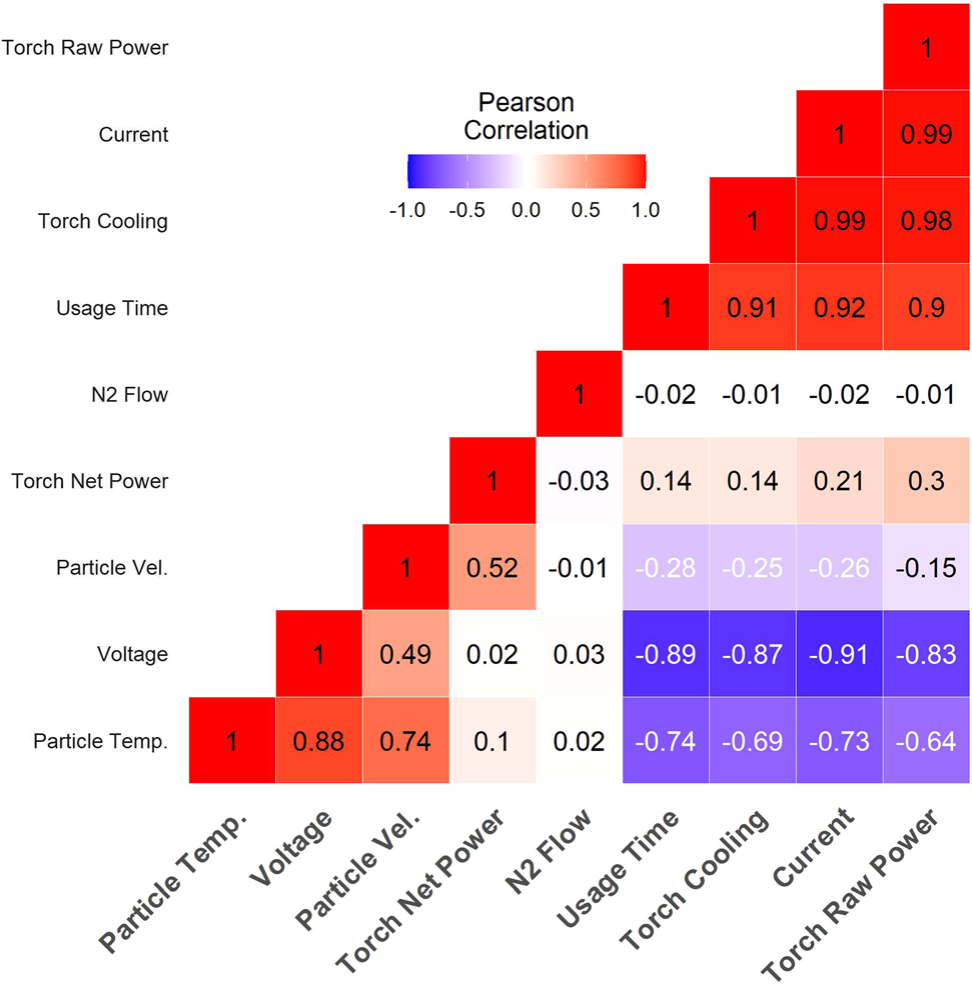
Pearson correlation coefficient among some selected parameters

Figure 4 shows the Pearson correlation coefficient among some selected parameters. The Pearson correlation coefficient is a measure of linear correlation between two parameters. It is determined by dividing the covariance of the two parameters by the product of their standard deviations. This normalized covariance only gives values from −1 to 1. When it is 1, the two parameters relate to each other linearly in a positive manner; increasing one, the other will increase too. When it is −1, the two parameters relate to each other linearly negatively; increasing one parameter, the other will decrease. As with the covariance, the Pearson correlation coefficient can only reflect if the two parameters have any linear relationship. Recall that in the experiment, only a single spray condition is considered. Several process parameters (e.g. flow rate of nitrogen) were not varied throughout the experiment. Therefore, their effects on the particle velocity and temperature cannot be observed and appear to have no relation to any other parameter. Similar observation is found for the net power, which is purposefully maintained constant. In particular, the Pearson correlation coefficient indicates that the particle temperature seems to have stronger correlation with the voltage as compared to particle velocity.

A time series has a temporal relationship among the observations (i.e. records): the previous observation affects the current one, which will affect the next one, etc. However, most machine learning predictive models, including RF and GB, consider each observation independently and thus, expect that all the necessary information to predict the target can be found in the attributes of that particular observation. Therefore, to forecast a time series using these machine learning models, it is necessary to capture the temporal order information properly as new attributes in each observation itself. There are two general approaches (Ref 26). The first approach simply appends all the information from the considered previous time segments into the observation (Ref 27). The time series may also be normalized (Ref 28), stationarized (Ref 17), or decomposed (Ref 30) a priori. The second approach only supplements the observation with specifically engineered and selected statistical features from the time series for the learning (Ref 26). The first approach will be adopted here due to its simplicity. One disadvantage of this approach, however, is that the number of the attributes increases rather quickly as longer histories are desired, resulting in a higher model training cost. Therefore, it is desirable to include only the relevant parameters for the attribute augmentation.

### Feature Selection

Both RF and GB provide a ranking of variable importance as the regression models are developed. They will be used to guide the useful attribute selection.

The present prediction problem belongs to that of multi-target regression, having two outputs: the particle temperature and velocity. The development of multi-target regression for RF is more advanced (Ref 31). There are already several implementations available from popular machine learning platforms (Ref 32, 33). Such models also take into consideration the correlation among the targets. As for GB, multi-target regression is still under active development. It is not yet possible to develop native multi-target regression using GB. A simple workaround is to build a separate predictive model per target. The correlation among the outputs is, however, ignored. Alternatively one may build a set of chained dependent models: Suppose that there are two targets ${y}_{a}$ and ${y}_{b}$. One first builds a model to predict ${y}_{a}$ with the attributes $\mathop{x}\limits^{\rightharpoonup} = x_{1} ,x_{2} , \ldots ,x_{n}$; and then one builds another model to predict ${y}_{b}$ with $\widehat{{y}_{a}}$ (the predicted ${y}_{a}$ from the first model) as well as the attributes $\mathop{x}\limits^{\rightharpoonup}$. The performance of such approach depends upon the model construction order and the correlation among the targets. The benefit of the added complexity is not always apparent.

Both ensemble methods will be used here to compare the features. The dataset is first randomly separated into two parts: 70% for training and 30% for testing.

The testing set is used to ensure the decent performance of the predictive model. The hyperparameters of the two models are set based on previous experience as follows: for RF, the maximum number of features per tree is set to 2 and the depth of tree is left as maximum; for GB, the depth of tree is set to 2, with a learning rate of 0.1. Here, the simpler separate model per target approach is employed. For both methods, a minimal leaf node size of 5 is considered.

**Fig. 5:**
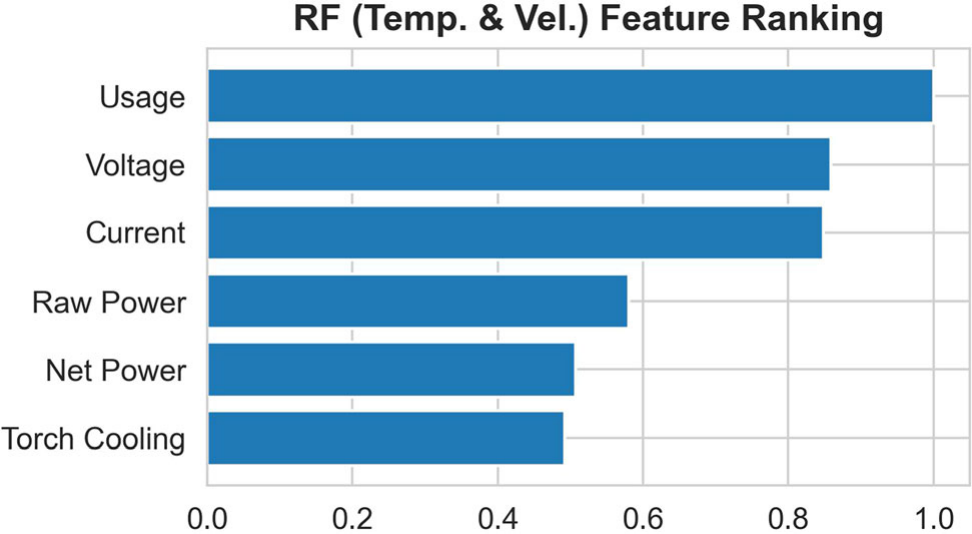
Top 6 features ranked using RF, which is trained to predict both the particle temperature and velocity simultaneously

**Fig. 6:**
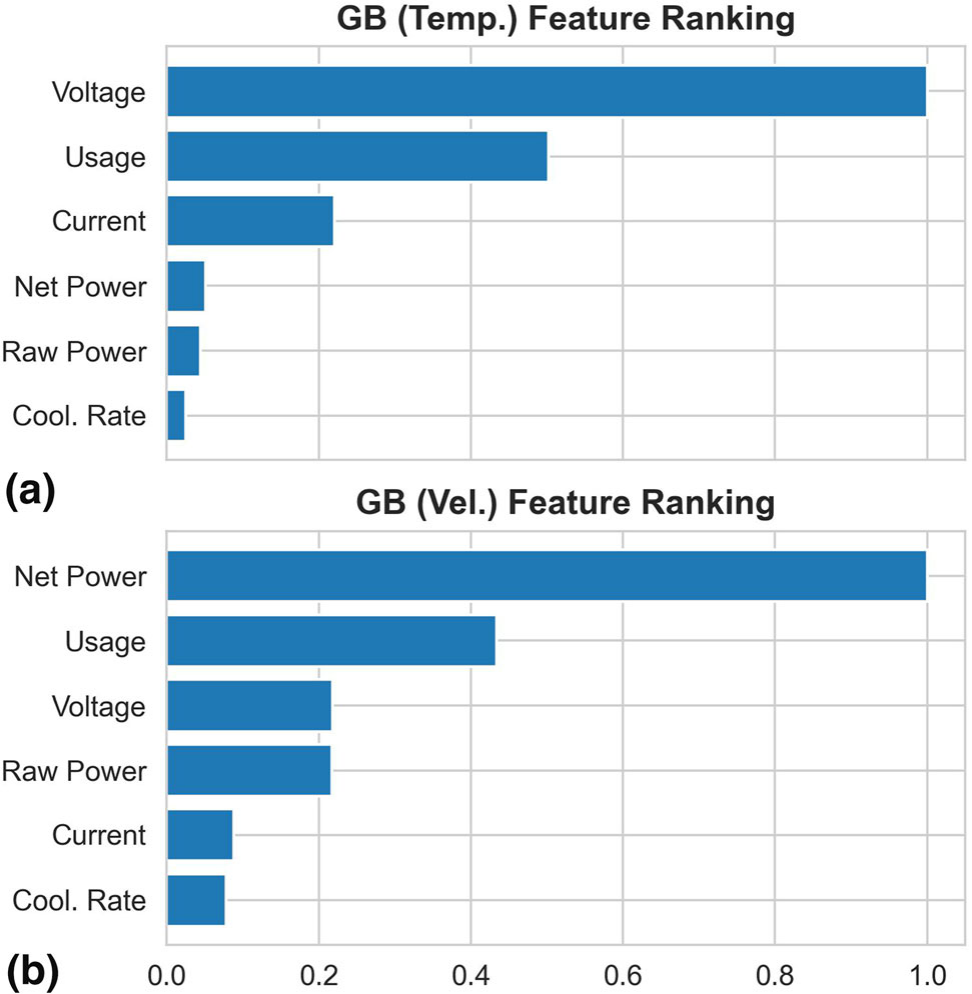
Top 6 features ranked using GB. One separated model is trained per target. (a) shows the feature ranking of the GB model trained to predict the particle temperature, and in (b), from the one to predict the particle velocity

The top 6 features ranked using RF and GB are shown in Fig. 5 and 6 respectively. The two sets of ranking are not identical, and both do not follow exactly the Pearson correlation coefficient (Fig. 4). The ranking of GB seems to have better agreements with the Pearson correlation coefficient than those of RF.

The ranking from GB for the in-flight particle velocity, though reasonable according to the Pearson correlation coefficient, is counterintuitive. In the ranking, the net power is the most important feature for the particle velocity prediction. From Fig. 4, indeed the net power is the most correlated feature with respect to the particle velocity after the particle temperature. However, the net power is purposefully maintained constant throughout the experiment. This underlines the importance of having the interaction among the targets considered. Therefore, in the following, only RF will be studied with the top 5 features ranked in Fig. 5. In particular, the torch net power is not included, because first, the ranking between the torch net power and the torch cooling are very comparable, and secondly the torch net power can be determined by simply subtracting the torch cooling from the torch raw power. It is not necessary to include redundant information.

### Time Series Attribute Augmentation

To incorporate the temporal order of the data in each observation, it is necessary to add the information of the previous observations into the present observation as new attributes. Suppose that there are two attributes: ${x}_{a}$ and ${x}_{b}$ in the original dataset for a target $y$. Embedding such dataset with the data from the two previous time steps will result in the following form:

$$\begin{array}{c}{{\varvec{y}}}_{3}\\ {{\varvec{y}}}_{4}\\ {{\varvec{y}}}_{5}\end{array} \Leftarrow \begin{array}{ccc}{{\varvec{x}}}_{{\varvec{a}}3}& {{\varvec{x}}}_{{\varvec{b}}3}& {y}_{2}\\ {{\varvec{x}}}_{{\varvec{a}}4}& {{\varvec{x}}}_{{\varvec{b}}4}& {y}_{3}\\ {{\varvec{x}}}_{{\varvec{a}}5}& {{\varvec{x}}}_{{\varvec{b}}5}& {y}_{4}\end{array} \begin{array}{cc}{x}_{a2}& {x}_{b2}\\ {x}_{a3}& {x}_{b3}\\ {x}_{a4}& {x}_{b4}\end{array} \begin{array}{ccc}{y}_{1}& {x}_{a1}& {x}_{b1}\\ {y}_{2}& {x}_{a2}& {x}_{b2}\\ {y}_{3}& {x}_{a3}& {x}_{b3}\end{array}$$

where the numbers represent the ascending temporal order in the dataset. The original dataset is shown in bold. Note that the target values for the previous observations are also embedded here. For example, ${x}_{a}$ can be the torch current intensity $I$ and ${x}_{b}$ can be the torch voltage $V$ with $y$ being the in-flight particle temperature ${T}_{p}$. The embedded dataset, again considering the two previous time steps, will become as follows:

$$\begin{array}{*{20}c} {{\varvec{T}}_{{{\varvec{p}}3}} } \\ {{\varvec{T}}_{{{\varvec{p}}4}} } \\ {{\varvec{T}}_{{{\varvec{p}}5}} } \\ \end{array} \Leftarrow \begin{array}{*{20}c} {{\varvec{I}}_{3} } & {{\varvec{V}}_{3} } & {T_{p2} } \\ {{\varvec{I}}_{4} } & {{\varvec{V}}_{4} } & {T_{p3} } \\ {{\varvec{I}}_{5} } & {{\varvec{V}}_{5} } & {T_{p4} } \\ \end{array} \begin{array}{*{20}c} {I_{2} } & {V_{2} } \\ {I_{3} } & {V_{3} } \\ {I_{4} } & {V_{4} } \\ \end{array} \begin{array}{*{20}c} {T_{p1} } & {I_{1} } & {V_{1} } \\ {T_{p2} } & {I_{2} } & {V_{2} } \\ {T_{p3} } & {I_{3} } & {V_{3} } \\ \end{array}$$

For a given dataset, incorporating a longer history can introduce more temporal information into the present observation. However, this will also reduce the total number of embedded observations for modeling. The tradeoff is far from trivial, and often the necessary lag order is problem-dependent. For the present exploration, the information (with also the targets) from the previous 4-time steps are embedded into the dataset. All the top 5 ranked features are included, resulting in 33 attributes in total in the embedded dataset, referred to as the simple embedded dataset in the following.

During data exploration, it is observed that both the targets and the attributes contain trending patterns. Hence, they are non-stationary. Traditional time series forecasting techniques will typically first use differencing to stationarize the time series data before model tuning (Ref 34). The benefits of such data preparation for RF is however not well reported. Therefore, a second embedded dataset, where the time series is first differenced before embedding, is prepared for comparison. This is referred to as the differenced embedded dataset below. Note that the prediction of any regression model, which is trained upon a differenced embedded dataset, requires reverse differencing to convert itself back to the original unit scale.

### Methodology for Forecasting Experiment

A RF regression model is developed from each embedded dataset. They are compared with a baseline RF model developed from the original dataset, without embedding nor differencing. Since the number of attributes ($m$) of these datasets is different, the number of features included per tree is set based on the rule of thumb recommended ($m/3$) (Ref 35), whereas the depth of tree is left as maximum as before with the minimum leaf node size of 5.

Again, the dataset is separated into two parts, according to each AccuraSpray measurement record: the beginning 70% for training and the remaining 30% for testing. This arrangement emphasizes the prediction of future values.

In this exploratory work, only the one-step prediction, which predicts the immediate next target value, is considered. The performance is compared using the mean square error (MSE) in the original unit scale.

## Results

### General Performance and Ensemble Sizing

**Fig. 7:**
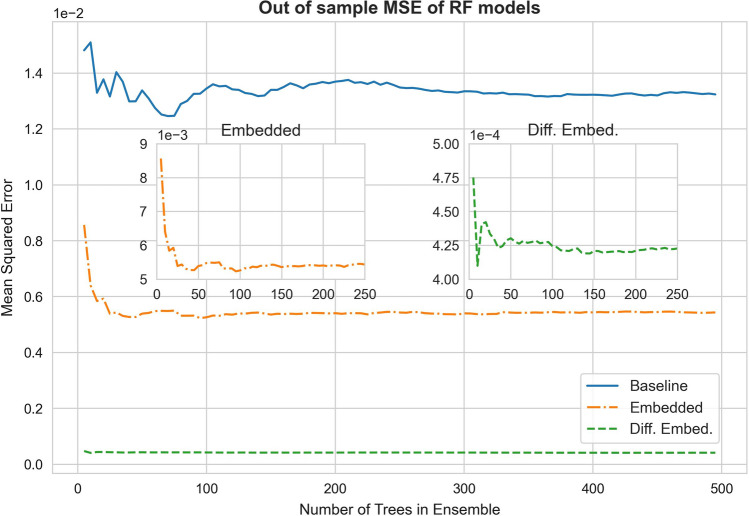
The out-of-sample mean squared error versus the number of trees in the ensemble of the three RF models

For each dataset, a set of RF regression models is first constructed, with an increasing number of trees from 5 to 500. Since the in-flight particle characteristic data captures the effect of electrode ageing, the prediction error is a direct indication of how well each model can cope with the aspect of electrode usage time. The corresponding out-of-sample MSE (i.e. computed from the testing data) versus the number of trees considered for the three RF model sets are shown in Fig. 7. The MSE of the simple embedded RF model is much smaller than that of the baseline model; whereas that of the differenced embedded RF model is substantially further reduced. For all three cases, the MSE reduces as the number of trees increases from the beginning. After a certain point, a further increase in the number of trees does not reduce the MSE anymore. This transition to the error plateau provides a guideline of the required number of trees for the ensemble as per the selected hyperparameters. From Fig. 7, it is indicated that the baseline RF model needs about 300 trees. Both the simple embedded and differenced embedded RF models (upon zoomed in) need about 200 trees.

Table 1 lists the in-sample MSE (i.e. computed from the training data) together with the out-of-sample MSE (i.e. computed from the testing data) for the three RF models. When the out-of-sample error is much higher than those of the in-sample, it is an indication of model over-fitting (i.e. when the model starts to capture also the noisy patterns in the training dataset, in addition to the overall trends as originally intended). The results from Table 1 suggest that the embedding procedure reduces model over-fitting. In particular, the differencing preprocessing essentially “eliminates” model over-fitting with respect to the present choice of hyperparameters.

**Table 1:** In-sample and out-of-sample mean square error (MSE) for the three RF models. The closer the out-of-sample error (computed from the testing data) to those of the in-sample (computed from the training data), the less model over-fitting exists

MSE	In-sample	Out-of-sample
Baseline	0.00056	0.01324
Simple Embed	0.00150	0.00543
Diff. Embed	**0.00048**	**0.00042**

Bold values indicate the superior performance of the Diff. Embed RF model

### Important Features

**Fig. 8:**
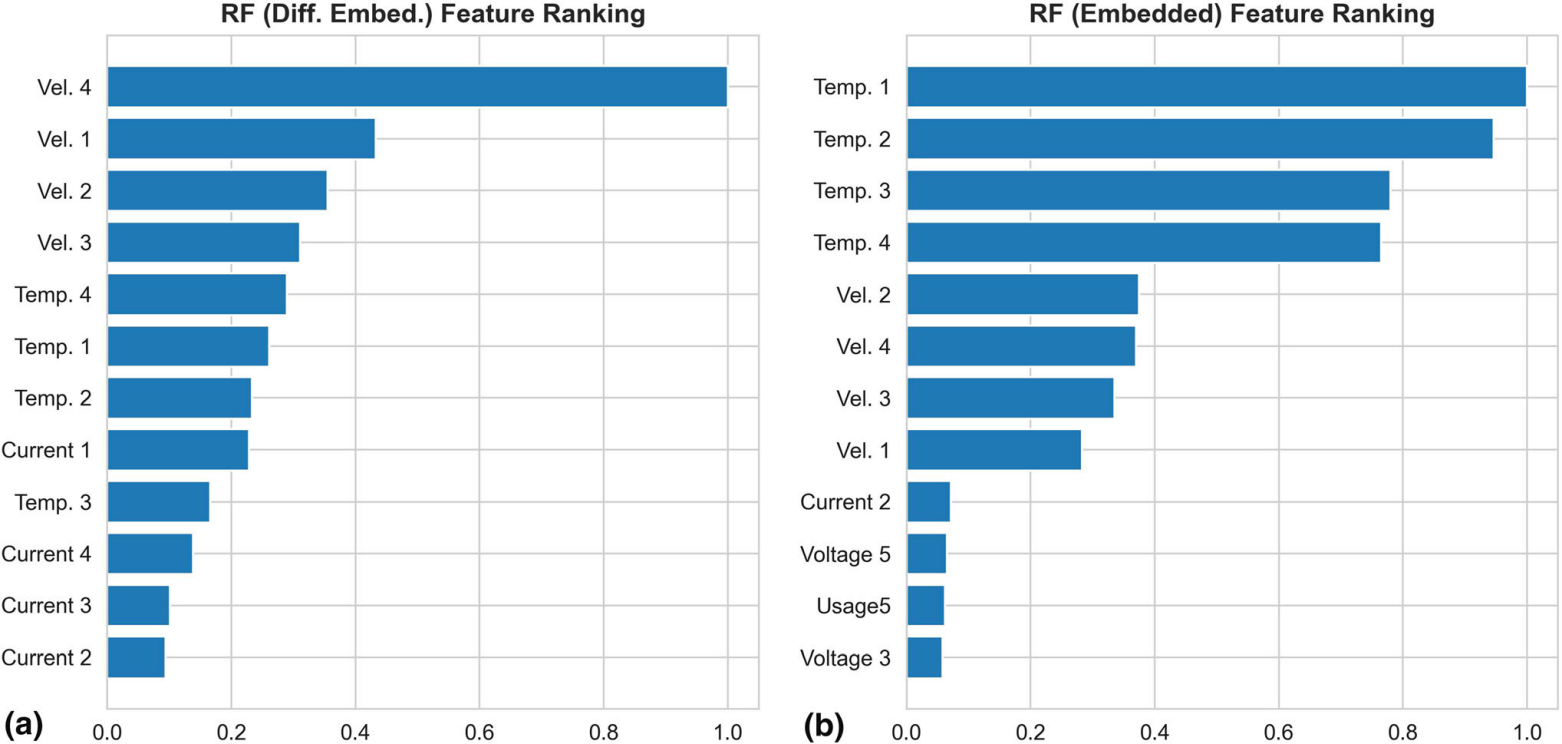
Rankings of features for differenced embedded (a) and simple embedded (b) RF models

As mentioned above, RF provides a ranking of variable importance as the models are developed. The feature ranking order of the baseline RF model is the same as Fig. 5. Whereas the top 12 features for the simple embedded and the differenced embedded RF models are shown in Fig. 8. The differenced embedded RF model ranks the previous targets (i.e. the previous particle temperatures and velocities) to be the most important attribute group, with a mixed order. After the previous targets, the model neatly ranks the torch currents, followed by the torch cooling, then the voltages, the raw powers and finally the electrode usage times. Whereas, the simple embedded RF model neatly ranks the previous particle temperature first, followed by particle velocity and the rest of the attributes in a mixed manner. The rankings from both embedded RF models underline the importance of the consideration of the previous target as attributes. Such consideration is not possible using the baseline RF model.

### Performance Comparison

The performance of a baseline RF model with 300 trees is then compared with two embedded RF models with 200 trees. Here, the tree depth is limited to 3 for all three RF models to better contrast their performance.

**Fig. 9:**
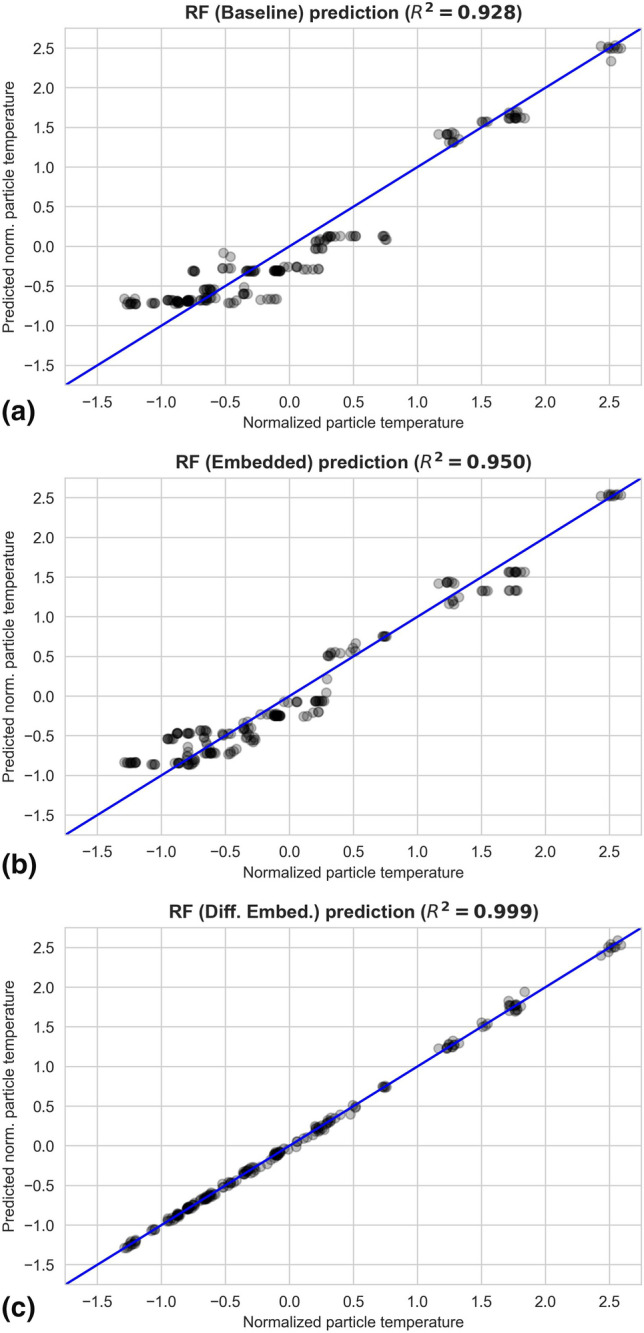
Comparison of the in-flight particle temperature predictions of the baseline (a), the simple embedded (b) and the differenced embedded (c) RF models. Note that all the attributes and the targets, including the in-flight particle temperature, are normalized by first subtracting the respective mean and then divided by their respective standard deviation

The comparison of the in-flight particle temperature prediction is shown in Fig. 9. The predictions are plotted against the actual values. The better the predictions are, the closer will they be with respect to the diagonal line. The scattering of the prediction reduces progressively from the baseline model (${R}^{2}=0.928$), then with the simple embedded RF model (${R}^{2}=0.950$), and finally becomes the smallest with the differenced embedded RF model (${R}^{2}=0.999$).

**Fig. 10:**
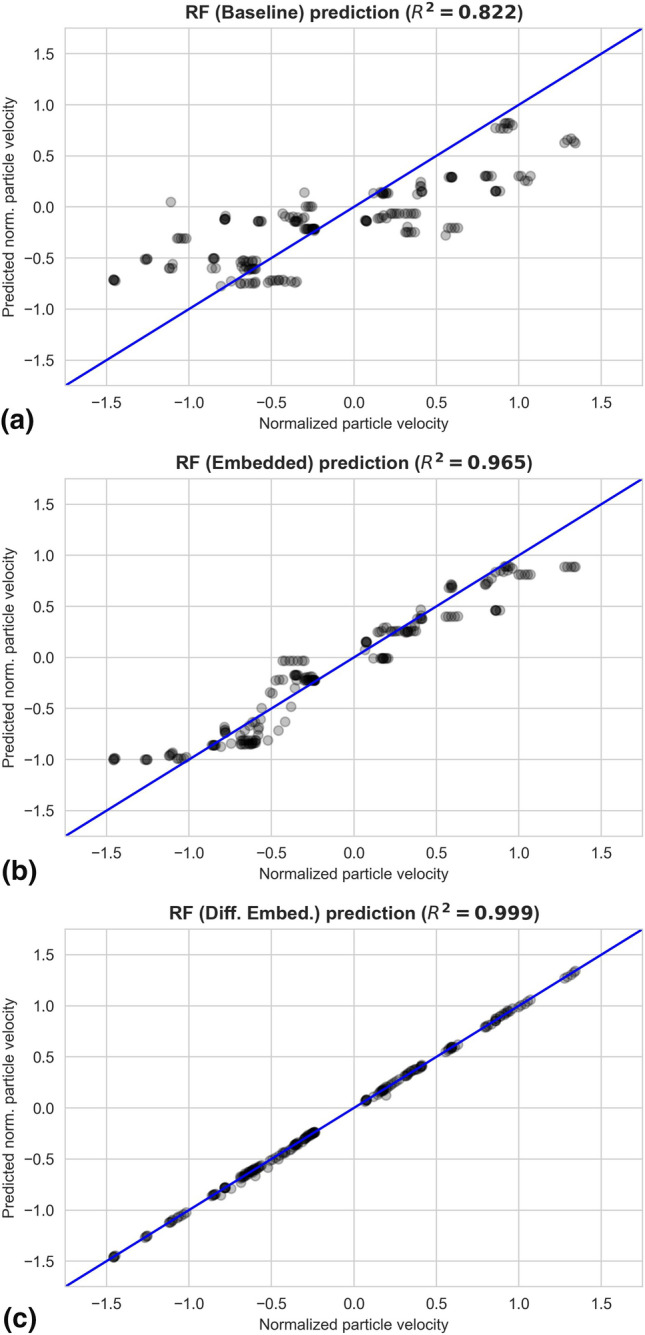
Comparison of the in-flight particle velocity predictions of the baseline (a), the simple embedded (b) and the differenced embedded (c) RF models. Note that all the attributes and the targets, including the in-flight particle velocity, are normalized by first subtracting the respective mean and then divided by their respective standard deviation

Figure 10 shows the comparison of the in-flight particle velocity prediction. Similar improvement trend can be observed, starting from the prediction of the baseline model (${R}^{2}=0.822$), then better with the simple embedded RF model (${R}^{2}=0.965$), and the best with the differenced embedded RF model (${R}^{2}=0.999$).

**Fig. 11:**
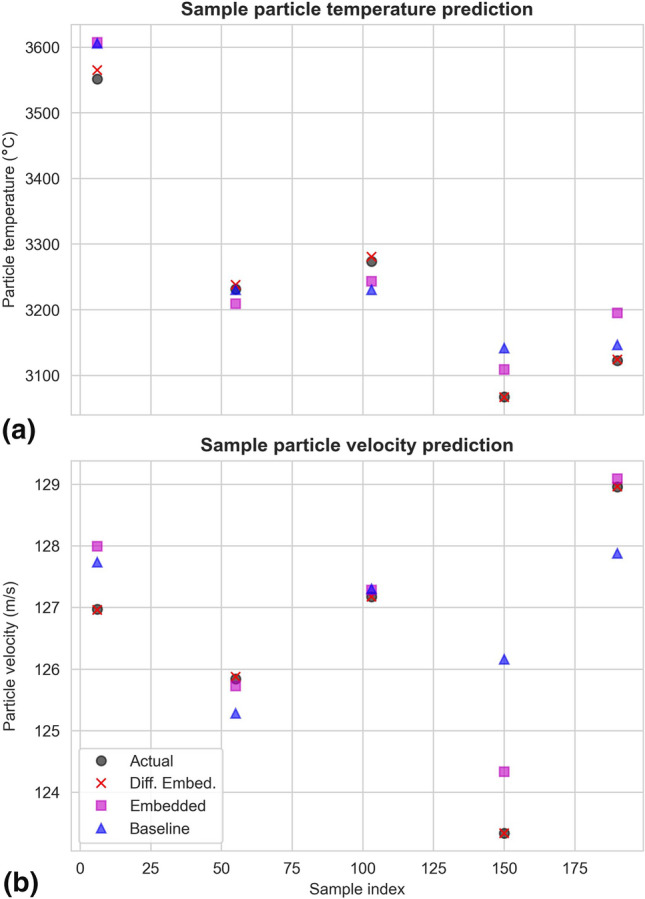
Selected predictions of the in-flight particle temperatures (a) and velocities (b) with the three RF models

Selected predictions of the in-flight particle temperatures and velocities are shown in Fig. 11. Interestingly, a few predictions of the baseline model are better than those of the simple embedded RF model here, e.g. the first velocity prediction on the left.

## Discussions

In literature, the majority of the predictive models proposed for APS in-flight particle characteristics are neural network based. The present investigation demonstrates that ensemble methods like RF are also suitable to model the APS in-flight particle characteristics. Compared with the neural network-based models, training a RF model is a lot easier. There are only a few hyperparameters (e.g. the number of feature included per tree, the depth of the tree, the minimum leaf node size, etc.) involved to specify the layout of an individual decision tree (Ref 36). The ‘forest’ can then be grown in a straight forward manner by building a new tree repeatedly from randomly sampled data with replacement. In contrast, to develop a neural network, one first needs to design the network structure by selecting the number of hidden layers, the number of neurons in each layer, their connection topology, and the activation function, etc. (Ref 37, 38) There is no specific rule of thumb for a successful neural network topology. Secondly, one needs to find the weighting parameters for the network components by solving a non-convex optimization problem; for example, using backpropagation (Ref 37, 38). It is not possible to compute an optimal set of the weights, nor there is any global convergence guarantees to find them. The process is generally computationally expensive. Besides, the complexity of the neural network dictates the minimum amount of data needed for its training. The present work now provides a straightforward alternative machine learning modeling approach for APS in-flight particle characteristics prediction.

The present work also underlines the benefits to consider the APS data as time series when modeling the in-flight particle characteristics since electrode ageing is of concern. The importance of such time evolving (i.e. time series) aspect of the in-flight particle characteristics as the electrode ages was not yet commonly acknowledged and was under appreciated in the corresponding predictive models. The majority of the predictive models proposed for APS in-flight particle characteristics do not consider the time evolution aspects of the process inputs parameters. Only a few articles consider such aspect. For instance, Liu et al. examined a time-series-sensitive nonlinear autoregressive exogenous (NLARX) model combined with the wavelet network to predict the in-flight particle characteristics of a mono-cathode plasma spray torch using a system identification approach (Ref 8). It was mentioned that such approach can better capture the dynamic characteristics, comparing with the normal neural network. Kim examined a linear autoregressive with exogenous (ARX) model and ARX-type neural network models to develop a control-oriented dynamic model of an inductively coupled plasma torch, similar to the work of Liu et al. (Ref 39). The present work uses RF to prepare three models: the baseline, the simple embedded and the differenced embedded. The latter two consider the in-flight particle characteristics as time series via the embedding procedures, which incorporates the data from the previous time steps into the present record, and they both have much improved performance over the baseline model for one-step prediction. In particular, it is highly beneficial to first make the time series stationary using the differencing technique. Since all three models are constructed using RF, the prediction improvement seen in those using the embedding procedures is offered by the datasets of these two models which contain information from the previous time steps. This provides clear supporting results to illustrate the benefits of considering the in-flight particle characteristics as time series over the lifespan of the electrodes.

Investigations are currently under way to evaluate other multivariate predictive models applicable for time series, as well as to compare the performance of the alternative time series enrichment strategy, which performs feature engineering and selection from the statistics of the time series (e.g. mean, standard deviation) for the learning. The corresponding findings will be communicated in future work. Although there are still knowledge and technological gaps before deploying a production-ready predictive model for the in-flight particle characteristics, the present study lays down a solid foundation to advance towards such goal.

## Conclusions

This work explored the applicability of two ensemble methods, namely RF and GB, to predict and forecast the multivariate APS in-flight particle characteristics with the consideration of torch electrodes ageing as time series. Experiments were performed to record simultaneously the input process parameters, the in-flight powder particle characteristics and the electrodes usage time, using a brand new pair of electrodes until they were no longer usable. Only a single spray condition was considered. The in-flight powder particle characteristics clearly reflect the effect of electrode ageing. Two strategies of time series embedding manipulation are considered. The first one simply stacks up the attributes and the targets from the previous $n$ time segments considered without any modification, while the second strategy first performs differencing to make the time series stationary before the embedding procedure. To select the more influencing attributes for the embedding procedure, an initial feature ranking is performed using both RF and GB. Such ranking underlines the advantages for those models that can consider the inter-target correlation for multivariate regression modeling. Hence, for the present application, RF is more suitable than GB. The superior prediction performances and the feature rankings of both embedded RF models show that it is better to consider the APS data as time series for the in-flight particle characteristic prediction. In particular, it is also advantageous to first make the time series stationary using the traditional differencing technique, even when modeling using RF. Comparison with other multivariate regression modeling techniques for time series is currently under way and the findings will be communicated in future work. Ultimately, it is desired to develop a predictive model for coating characteristics and performance, which can serve as a guiding tool for effective torch usage and coating quality control.

## References

[1] S. Guessasma, G. Montavon, P. Gougeon, and C. Coddet, Designing Expert System Using Neural Computation in View of the Control of Plasma Spray Processes, *Mater. Des.*, 2003, **24**(7), p 497-502. 10.1016/S0261-3069(03)00109-2

[2] S. Guessasma, G. Montavon, and C. Coddet, Modeling of the APS Plasma Spray Process Using Artificial Neural Networks: Basis, Requirements and an Example, *Comput. Mater. Sci.*, 2004, **29**(3), p 315-333. 10.1016/j.commatsci.2003.10.007

[3] A.-F. Kanta, M.-P. Planche, G. Montavon, and C. Coddet, In-Flight and Upon Impact Particle Characteristics Modelling in Plasma Spray Process, *Surf. Coat. Technol.*, 2010, **204**(9-10), p 1542-1548. 10.1016/j.surfcoat.2009.09.076

[4] W. Sha, Comment on Modelling of the APS Plasma Spray Process Using Artificial Neural Networks: Basis, Requirements and an Example’ by Guessasma et al. [Comput. Mater. Sci. 29 (2004) 315], *Comput. Mater. Sci.*, 2010, **50**, p 805-809. 10.1016/j.commatsci.2010.09.013

[5] T.A. Choudhury, N. Hosseinzadeh, and C.C. Berndt, Artificial Neural Network application for predicting in-flight particle characteristics of an atmospheric plasma spray process, *Surf. Coat. Technol.*, 2011, **205**(21-22), p 4886-4895. 10.1016/j.surfcoat.2011.04.099

[6] T.A. Choudhury, C.C. Berndt, and Z. Man, An Extreme Learning Machine Algorithm to Predict the In-flight Particle Characteristics of an Atmospheric Plasma Spray Process, *Plasma Chem. Plasma Process.*, 2013, **33**(5), p 993-1023. 10.1007/s11090-013-9466-4

[7] T.A. Choudhury, C.C. Berndt, and Z. Man, Modular Implementation of Artificial Neural Network in Predicting In-Flight Particle Characteristics of an Atmospheric Plasma Spray Process, *Eng. Appl. Artif. Intell.*, 2015, **45**, p 57-70. 10.1016/j.engappai.2015.06.015

[8] T. Liu, S. Deng, M.-P. Planche, and G. Montavon, Estimating the Behavior of Particles Sprayed by a Single-Cathode Plasma Torch Based on a Nonlinear Autoregressive Exogenous Model, *Surf. Coat. Technol.*, 2015, **268**, p 284-292. 10.1016/j.surfcoat.2014.10.040

[9] J. Zhu, X. Wang, L. Kou, L. Zheng, and H. Zhang, Prediction of Control Parameters Corresponding to In-Flight Particles in Atmospheric Plasma Spray Employing Convolutional Neural Networks, *Surf. Coat. Technol.*, 2020, **394**, 125862. 10.1016/j.surfcoat.2020.125862

[10] A.-F. Kanta, G. Montavon, C.C. Berndt, M.-P. Planche, and C. Coddet, Intelligent System for Prediction and Control: Application in Plasma Spray Process, *Expert Syst. Appl.*, 2011, **38**(1), p 260-271. 10.1016/j.eswa.2010.06.056

[11] T. Liu et al., Plasma Spray Process Operating Parameters Optimization Based on Artificial Intelligence, *Plasma Chem. Plasma Process.*, 2013, **33**(5), p 1025-1041. 10.1007/s11090-013-9475-3

[12] J. Zhu, X. Wang, L. Kou, L. Zheng, and H. Zhang, Application of Combined Transfer Learning and Convolutional Neural Networks to Optimize Plasma Spraying, *Appl. Surf. Sci.*, 2021, **563**, 150098. 10.1016/j.apsusc.2021.150098

[13] S. Guessasma, G. Montavon, and C. Coddet, Analysis of the Influence of Atmospheric Plasma Spray (APS) Parameters on Adhesion Properties of Alumina-Titania Coatings, *J. Adhes. Sci. Technol.*, 2004, **18**(4), p 495-505. 10.1163/156856104323016388

[14] L. Wang, J.C. Fang, Z.Y. Zhao, and H.P. Zeng, Application of Backward Propagation Network for Forecasting Hardness and Porosity of Coatings by Plasma Spraying, *Surf. Coat. Technol.*, 2007, **201**(9-11), p 5085-5089. 10.1016/j.surfcoat.2006.07.088

[15] A.-F. Kanta, G. Montavon, M.-P. Planche, and C. Coddet, Artificial Intelligence Computation to Establish Relationships Between APS Process Parameters and Alumina-Titania Coating Properties, *Plasma Chem. Plasma Process.*, 2008, **28**(2), p 249-262. 10.1007/s11090-007-9116-9

[16] Z. Wu, Empirical Modeling for Processing Parameters’ Effects on Coating Properties in Plasma Spraying Process, *J. Manuf. Process.*, 2015, **19**, p 1-13. 10.1016/j.jmapro.2015.03.007

[17] C.-M. Lin, S.-H. Yen, and C.-Y. Su, Measurement and Optimization of Atmospheric Plasma Sprayed CoMoCrSi Coatings Parameters on Ti-6Al-4V Substrates Affecting Microstructural and Properties Using Hybrid Abductor Induction Mechanism, *Measurement*, 2016, **94**, p 157-167. 10.1016/j.measurement.2016.07.077

[18] M. Kuhn and K. Johnson, *Applied Predictive Modeling*, Springer, New York, 2013.

[19] R. Caruana and A. Niculescu-Mizil, An empirical comparison of supervised learning algorithms, In: *Proceedings of the 23rd international conference on Machine learning - ICML ’06*, Pittsburgh, Pennsylvania, 2006, pp. 161-168. doi: 10.1145/1143844.1143865.

[20] M. Fernández-Delgado, E. Cernadas, S. Barro, and D. Amorim, Do we Need Hundreds of Classifiers to Solve Real World Classification Problems?, *J. Mach. Learn. Res.*, 2014, **15**(90), p 3133-3181.

[21] L. Breiman, Random Forests, *Mach. Learn.*, 2001, **45**(1), p 5-32. 10.1023/A:1010933404324

[22] J.H. Friedman, Greedy Function Approximation: A Gradient Boosting Machine, *Ann. Stat.*, 2001, **29**(5), p 1189-1232. 10.1214/aos/1013203451

[23] J.H. Friedman, Stochastic Gradient Boosting, *Comput. Stat. Data Anal.*, 2002, **38**(4), p 367-378. 10.1016/S0167-9473(01)00065-2

[24] B. Boehmke and B.M. Greenwell, *Hands-on Machine Learning with R*, CRC Press, Boca Raton, 2019.

[25] D.H. Wolpert, The Lack of A Priori Distinctions Between Learning Algorithms, *Neural Comput.*, 1996, **8**(7), p 1341-1390. 10.1162/neco.1996.8.7.1341

[26] M. Hostetter, A. Ahmadzadeh, B. Aydin, M. K. Georgoulis, D. J. Kempton, and R. A. Angryk, Understanding the Impact of Statistical Time Series Features for Flare Prediction Analysis, In: *2019 IEEE International Conference on Big Data (Big Data)*, Los Angeles, CA, USA, 2019, pp. 4960-4966. doi: 10.1109/BigData47090.2019.9006116.

[27] E. Mussumeci and F. Codeço Coelho, Large-Scale Multivariate Forecasting Models for Dengue LSTM Versus Random Forest Regression, *Spatio-Temporal Epidemiol Spat*, 2020 10.1016/j.sste.2020.100372 10.1016/j.sste.2020.100372 33138951

[28] G. Dudek, Short-Term Load Forecasting Using Random Forests, In: *Intelligent Systems’2014*, vol. 323, D. Filev, J. Jabłkowski, J. Kacprzyk, M. Krawczak, I. Popchev, L. Rutkowski, V. Sgurev, E. Sotirova, P. Szynkarczyk, and S. Zadrozny, Eds. Cham: Springer International Publishing, 2015, pp. 821-828. doi: 10.1007/978-3-319-11310-4_71.

[29] C. Paoli, C. Voyant, M. Muselli, and M.-L. Nivet, Forecasting of Preprocessed Daily Solar Radiation Time Series Using Neural Networks, *Sol. Energy*, 2010, **84**(12), p 2146-2160. 10.1016/j.solener.2010.08.011

[30] P.-H. Chiang, S. P. V. Chiluvuri, S. Dey, and T. Q. Nguyen, Forecasting of Solar Photovoltaic System Power Generation Using Wavelet Decomposition and Bias-Compensated Random Forest, In: *2017 Ninth Annual IEEE Green Technologies Conference (GreenTech)*, Denver, CO, USA, 2017, pp. 260-266. doi: 10.1109/GreenTech.2017.44

[31] M. Segal and Y. Xiao, Multivariate Random Forests, *WIREs Data Min. Knowl. Discov.*, 2011, **1**(1), p 80-87. 10.1002/widm.12

[32] Comparing random forests and the multi-output meta estimator — scikit-learn 0.24.1 documentation. https://scikit-learn.org/stable/auto_examples/ensemble/plot_random_forest_regression_multioutput.html (accessed 19, 2021).

[33] H. Ishwaran and U. B. Kogalur, *randomForestSRC: Fast Unified Random Forests for Survival, Regression, and Classification (RF-SRC)*. 2020. Accessed: 19, 2021. [Online]. Available: https://CRAN.R-project.org/package=randomForestSRC

[34] R. J. Hyndman and G. Athanasopoulos, Forecasting: Principles and Practice (3rd ed). Accessed: 10, 2021. [Online]. Available: https://Otexts.com/fpp3/

[35] T. Hastie, R. Tibshirani, and J.H. Friedman, *The Elements of Statistical Learning: Data Mining, Inference, and Prediction*, 2nd ed. Springer, New York, NY, 2009.

[36] sklearn.ensemble.RandomForestRegressor, *scikit-learn*. https://scikit-learn/stable/modules/generated/sklearn.ensemble.RandomForestRegressor.html (accessed 08, 2022).

[37] J. Brownlee, A Gentle Introduction to the Challenge of Training Deep Learning Neural Network Models,” *Machine Learning Mastery*, 14, 2019. https://machinelearningmastery.com/a-gentle-introduction-to-the-challenge-of-training-deep-learning-neural-network-models/ (accessed 08, 2022).

[38] A Recipe for Training Neural Networks. http://karpathy.github.io/2019/04/25/recipe/ (Accessed 08, 2022).

[39] K.S. Kim, Control-Oriented Dynamic Model of an Inductively Coupled Plasma Torch by Artificial Intelligence Methodology, *Plasma Phys. Control. Fusion*, 2019, **61**(4), 044002. 10.1088/1361-6587/aaffb4

